# Social Factors, Dietary Intake and the Nutritional Status of Community-Dwelling Chinese Older Adults: A Scoping Review

**DOI:** 10.3390/nu17122019

**Published:** 2025-06-17

**Authors:** Joyce P. Y. Tsang, Daphne S. K. Cheung, Justina Y. W. Liu

**Affiliations:** 1 S.K. Yee Department of Health Sciences, Saint Francis University, Hong Kong, China; 2 School of Nursing, The Hong Kong Polytechnic University, Hong Kong, China; d.cheung@deakin.edu.au (D.S.K.C.); justina.liu@polyu.edu.hk (J.Y.W.L.); 3 School of Nursing and Midwifery, Deakin University, Burwood, VIC 3125, Australia; 4 Institute for Smart Ageing, The Hong Kong Polytechnic University, Hong Kong, China; 5 Research Centre for Assistive Technology, The Hong Kong Polytechnic University, Hong Kong, China

**Keywords:** malnutrition, social factors, social frailty, Chinese, older adult, scoping review

## Abstract

**Background/Objectives:** Malnutrition can lead to poor health outcomes and mortality. Older adults are at a high risk of malnutrition due to age-related changes in their body and their dietary intake. The dietary intake of community-dwelling older adults can be affected by material and biopsychosocial factors. Conventional interventions often omit the influence of social factors on dietary intake—a particularly significant omission in the Chinese culture which sees eating as a social affair. This scoping review aimed to identify and understand the social factors associated with the dietary intake or nutritional status of community-dwelling Chinese older adults. **Methods:** This scoping review followed stages of research question identification, studies identification and selection, data charting, and results reporting. A systematic search was conducted in December 2024 for primary studies from databases, and reference lists of review articles were screened. Data extracted included characteristics of the study, measures of nutritional status, measures of social factors, and key findings. **Results:** A total of 964 articles were identified. Twelve studies were included in the review. Five social factors were identified as associated with dietary intake or nutritional status: (1) marital status; (2) living arrangement; (3) eating arrangement; (4) loneliness, social support and social isolation; and (5) social frailty. Being single, eating alone, experiencing loneliness or isolation, and being socially frail were found to be associated with poorer dietary intake or nutritional status, though the impact of living alone remains inconclusive. **Conclusions:** The relationship between social factors and dietary intake or nutritional status has not been extensively studied. Among the factors identified, it was found that eating arrangement and social frailty are potentially modifiable. Interventions targeting these social aspects could be developed.

## 1. Introduction

Malnutrition in the literature commonly refers to undernutrition, but it includes all forms of poor nutrition, which includes undernutrition, overnutrition, and nutrient deficiency. Older adults are at a high risk of malnutrition due to age-related changes in body composition, body functions, and dietary intake [1]. Both sides of suboptimal nutritional status can lead to increased morbidity and mortality. The impacts of undernutrition in older adults include a poor clinical outcome from disease, trauma and surgery, skeletal muscle wasting, decreased bone mineral mass, impaired immune function, increased risk of geriatric syndromes, and poor quality of life [2]. On the other hand, overnutrition increases risks of cardiovascular diseases and cancer, and mobility issues [3]. These pose a great burden on the health care system.

Community-dwelling older adults usually have better well-being than institutionalized older adults. However, meals are provided for institutionalized people, while community-dwelling older adults must attend to their own diet. A recent meta-analysis on the nutritional status of older people in different settings, which used the Mini Nutritional Assessment (MNA) as a screening tool, revealed that 5–11% of older adults living in the community are undernourished, and 31–40% of them are at risk of undernutrition [4]. For overnutrition, a systematic review and meta-analysis with studies mostly on community-dwelling older adults concluded the prevalence of obesity (BMI ≥ 30) is around 25% globally [5].

Multiple factors could contribute to the nutritional intake of community-dwelling older adults. Material (tangible resources), physical, psychological, and social factors can affect different processes involved in food intake, including the purchase, preparation, and consumption of food [6]. Conventional interventions for malnourished older adults target different factors of nutritional intake. For example, medical treatments [7] for diseases target physical and psychological factors involved in the purchase, preparation, and consumption of food. Receiving dentures from a dentist can resolve problems of dentition, which is a material factor in the consumption of food. With regard to nutrition interventions, oral nutritional supplements can also resolve a material factor involved in the consumption of food [8]. Dietetic counselling can improve psychological factors in the preparation and consumption of food in relation to knowledge, beliefs, and attitudes [7,8]. Food texture modification, as in a prescribed diet, can help with dietary intake for people who experience problems with chewing and swallowing, which are physical factors affecting the consumption of food [7]. Lastly, food and other welfare services target social factors involved in the purchase of food, as well as material factors in the purchase and preparation of food [8]. However, these conventional interventions have failed to address many of the social factors associated with the dietary intake of older adults. These social factors include broader concepts such as social support, social networks, living arrangements, and marital status; and specific aspects of dining experiences such as eating arrangements (the physical presence of someone else during meals), commensality (the act of eating together at the same table), and mealtime interactions (social interactions during meals) [9].

Some systematic reviews have explored social factors affecting malnutrition in older adults [9,10,11,12]. The majority of the studies included in these reviews have been from ‘modern Western’ cultures, with few to no studies on Chinese older adults. The few Chinese studies included in these reviews have been interpreted in aggregation with studies on other cultures. This might not take into consideration the cultural influence on how social factors affect health. A cross-cultural study showed that cultural individualism had a significant moderating effect on how loneliness influences health in general [13]. Specifically on nutrition, it was found that people from Western cultures that stress individualism, such as the United States, the United Kingdom, Germany, and Sweden, valued eating as an individual affair, with everyone having an autonomous choice of food [14]. Social aspects of eating were less essential as a component of a good meal in these cultures, but rather more correlated with festivities [15]. Eating alone in older adults in these cultures was seen as a routine and could be a sign of independence and contentment [16]. On the other hand, cultures that prioritize collectivism, such as African, Mediterranean, and Asian cultures, regard eating as an opportunity for social bonding [17,18,19,20]. In the Chinese culture, mealtimes tend to involve eating with relatives and friends sitting around a round table and sharing common dishes [21]. How social factors affect the dietary intake or nutritional status of Chinese older adults remains unclear. Therefore, the aim of this scoping review was to identify and understand the social factors associated with the dietary intake or nutritional status of community-dwelling Chinese older adults.

## 2. Methods

This was a scoping review that followed the stages of Arksey and O’Malley’s framework [22]: (1) identifying the research question; (2) identifying relevant studies; (3) selecting the studies; (4) charting the data; and (5) collating, summarizing, and reporting the results. The results were reported in alignment with the PRISMA checklist for scoping reviews [23] and the PRISMA 2020 flowchart for reporting systematic reviews [24].

*Stage 1: Identifying the research question*

To facilitate in-depth and broad results, open research questions were developed as follows:

1. What are the social factors relating to dietary intake or nutritional status among community-dwelling Chinese older adults?
2. How do the social factors relate to dietary intake or nutritional status among community-dwelling Chinese older adults?

*Stage 2: Identifying relevant studies*

A literature search was conducted in December 2024 on nursing, health science, and social science databases, including PubMed, CINAHL, Embase, Web of Science, China Academic Journal Network Publishing Database (CNKI), and Wanfang Database. The search terms that were used included synonyms for ‘community-dwelling’, ‘Chinese’, ‘aged’, ‘social factors’, ‘dietary intake’, and ‘nutritional status’. The search terms are presented in Table 1 and the syntax for the PubMed search strategy is attached in Table S1 as an example. No limit was set on the time of publication to enable a broad search to be conducted. Additional articles were also identified from the reference lists of review articles obtained from the database search. All articles were exported to EndNote 20, where duplicates were removed.

*Stage 3: Selecting the studies*

The inclusion and exclusion criteria were as follows: studies were included if (1) over 50% of the study population were aged 60 years or above, or the mean age of the study population was above 60 years; (2) the study population consisted of those living in the community; (3) over 50% of the study population was of Chinese ethnicity; (4) data on dietary intake or nutritional status were reported, including anthropometric measurements, biochemical markers, or measurements of nutritional risk; (5) the relationship between a social factor and dietary intake or nutritional status was explored; (6) the study was published in English or Chinese; (7) the study was observational or interventional. Excluded were case reports, study protocols, letters to editors, or review articles.

*Stage 4: Charting the data*

A data extraction form was adapted from the JBI Manual for Evidence Synthesis [25]. Extracted were data on the authors, year of publication, study location, study design, number and characteristics of the study population, measures of dietary intake/nutritional status, measures of social factors, and key findings relating to social factors and dietary intake/nutritional status.

*Stage 5: Collating, summarizing, and reporting the results*

An inductive thematic approach was used to collate and summarize the results. The characteristics of the studies, ranges of measures used, and the relationship of social factors and dietary intake/nutritional status were reported using narrative description.

The quality of the included studies was assessed using the corresponding JBI critical appraisal checklists for their study design [26]. Cross-sectional studies were assessed on the definition of the inclusion criteria, description of the study subjects and setting, measurements of exposure, condition, and outcome; identification and control of confounders, and appropriateness of the statistical analysis. Cohort studies were assessed on the recruitment of subjects, measurements of exposure and outcome, identification and control of confounders, sufficiency of the follow-up time, reporting of drop-outs, strategies to address incomplete follow-ups, and appropriateness of the statistical analysis. The quality of the studies was reported in terms of the percentage of ‘yes’ responses in meeting the applicable assessment criteria. A higher percentage indicated a higher quality and a lower risk of bias.

## 3. Results

**Figure 1:**
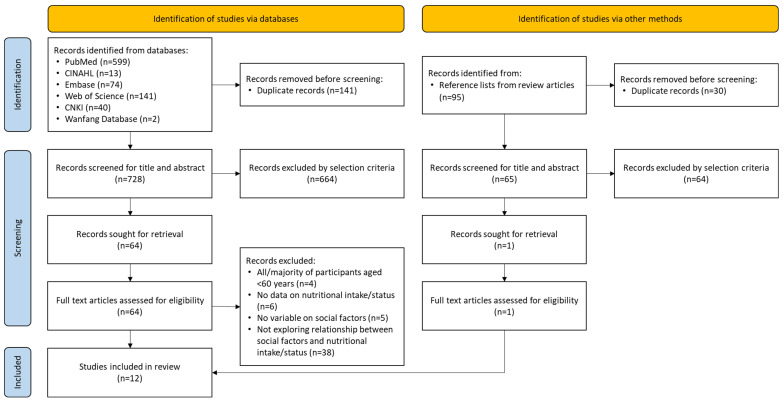
Flowchart of the literature selection process.

The search of the databases identified 869 articles. An additional 95 articles were identified from the reference lists of review articles (Figure 1). One hundred and seventy-one duplicate articles were removed. The titles and abstracts of 793 articles were screened against the inclusion and exclusion criteria. Subsequently, 728 articles were excluded mainly because they did not explore any social factors. The full texts of the remaining 65 articles were extracted and assessed for eligibility. Fifty-three were excluded for not meeting the inclusion criteria, for example, the majority of the participants were not older adults, no social factors were explored, no nutritional data were reported, or the social factors and nutritional data were studied separately. In the end, 12 articles were included in this scoping review [23,24,25,26,27,28,29,30,31,32,33,34].

### 3.1. Characteristics of the Studies

The included studies were conducted in China (*n* = 9) [27,28,29,30,31,32,33,34,35], Singapore (*n* = 2) [36,37], and Taiwan (*n* = 1) [38] from 2007 to 2024 (Table 2). Two were cohort studies and the remainder were all observational cross-sectional studies. Dietary intake/nutritional status was the outcome measure in eight studies, while social factors were the outcome measure in two studies. For the remaining two studies, the outcome measures were mortality and quality of life, but relationships between social factors and nutritional intake were also reported.

### 3.2. Measures Used in the Included Studies

Dietary intake/nutritional status was measured by (1) the quantity of the dietary intake, using an instrument such as the Food Frequency Questionnaire (FFQ) [38], the consumption of breakfast [31] and the state of the individual’s appetite [28]; (2) the quality of the dietary intake, through an instrument such as the Diet Diversity Score (DDS) [38]; (3) anthropometric measurements, such as muscle mass [27,30], body mass index (BMI) [31,33,38], and waist circumference (WC) [31,33]; (4) biomarkers, such as serum albumin levels [36]; and (5) malnutrition risk, using measurements such as the MNA [29,36], the European Society of Parenteral and Enteral Nutrition and Metabolism (ESPEN)’s definition of malnutrition [35], DETERMINE [37], and the Risk Assessment of Malnutrition in the Elderly [32,34].

Social factors were measured by (1) marital status [29,32,35,37]; (2) living arrangement [28,29,32,37]; (3) eating arrangement [38]; (4) loneliness, social support, and social isolation, through instruments such as the UCLA Loneliness Scale [28,33], the Social Support Rating Scale [30], or the Lubben Social Network Scale [34]; and (5) social frailty [31,36].

### 3.3. Quality of the Studies

The included studies had quality scores ranging from 87.5 to 100% (Table 3 and Table 4). Only two out of the 12 included studies failed to fulfil all of the criteria. One study included a questionnaire on social frailty with questions that had been adapted from three different sources without any indication that a process of validation had been conducted [36], while the other study failed to clearly define the criteria for the selection of the subjects [33].

### 3.4. Relationships Between Social Factors and Dietary Intake/Nutritional Status

#### 3.4.1. Marital Status

Table 5 presents a summary of the relationships between social factors and dietary intake or nutritional status. Among the included studies, marital status was the most reported variable in terms of social factors. Marital status was analyzed dichotomously, with married participants comprising one group, and single, divorced, or widowed participants making up another group. The results indicated a correlation between widowed status and poorer nutritional status [29]. Single, divorced, or widowed participants were more likely to be malnourished [35] or to be at a higher risk of malnutrition [37].

#### 3.4.2. Living Arrangement

Categories of living arrangement were as follows living alone/with spouse/with children [29], or alone/with others [28,32,37]. One study found that living arrangement was not associated with nutritional status [29], while other studies found that participants who lived alone were more likely to be at a higher risk of malnutrition [28,32,37].

#### 3.4.3. Eating Arrangement

Eating arrangement was only reported in one study, and was defined as the daily frequency of eating with others [38]. Among female participants, the low frequency of eating with others was associated with low BMI, a less diverse diet, and a low intake of meat, seafood, eggs, and vegetables. For male participants, a low frequency of eating with others was associated with a less diverse diet, a higher intake of meat, and a lower intake of vegetables.

#### 3.4.4. Loneliness, Low Social Support, and Social Isolation

Loneliness was measured in two studies [28,33], while social support/isolation was reported in three other studies [27,30,34]. Loneliness or a lack of social support were related to low muscle mass [27,30], lower appetite (the anorexia of ageing) [28], and a lower BMI [33]. Social isolation was also found to be weakly associated with malnutrition risk [34].

#### 3.4.5. Social Frailty

The broader concept of social frailty was explored in two studies [31,36]. In a study by Pek et al. [36], social frailty was described with reference to a conceptual framework of social frailty by Bunt et al. [39], while it was described as living alone, having a lack of social relations, and a lack of social support in a study by Song et al. [31]. Bunt et al.’s conceptual framework suggests that social resources, social behaviour/activities, and general resources affect the fulfilment of basic social needs [39]. Social frailty was found to be correlated with poor nutritional status as measured by MNA [36], low serum albumin levels [36], no consumption of breakfast [31], overweight or obesity as measured by BMI [31], and central obesity as determined by waist circumference [31].

## 4. Discussion

This scoping review included 10 cross-sectional studies and two cohort studies, which only presented the associations between the social factors and nutritional status. No qualitative studies were found. Hence, no studies were found to explore the hows and whys of the relationship between social factors and dietary intake or nutritional status. Moreover, no interventional studies for the Chinese older population were found in this scoping review, which implies that no documented social interventions have been carried out to improve the dietary intake or nutritional status of this population. By contrast, various experimental studies have been conducted in other cultures. For example, a scoping review [40] identified three studies that used social facilitation as an intervention for older adults to increase their dietary intake [41,42,43], and two of those studies showed positive results in increasing the dietary intake of the participants [41,42].

**Figure 2:**
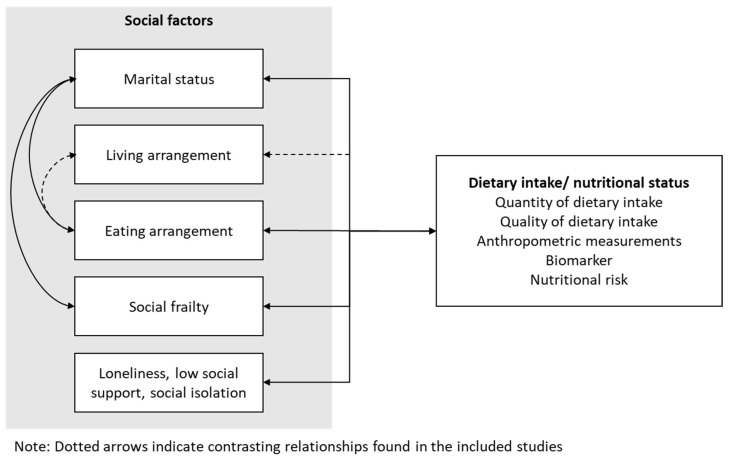
Relationships between social factors and dietary intake/ nutritional status.

The results showed that being single (including being divorced and widowed), eating alone, experiencing loneliness or isolation, and being socially frail were associated with poorer nutritional status or dietary intake, while living alone may or may not be associated. The findings were congruent with a few systematic reviews, including studies conducted in different countries [9,10,11,12]. Some of the included studies also explained the interaction among these social factors (Figure 2). For example, marital status affects eating arrangement and social frailty, as married older adults are more likely to eat with others [38], while single older adults scored higher in social frailty [31]. On the other hand, only around 50–60% of older adults who did not eat with others were living alone [38]. In other words, some older adults eat alone despite living with others. This might suggest that eating arrangement can be more influential than living arrangement on the dietary intake or nutritional status of Chinese older adults.

Loneliness, social support, and social isolation are related but slightly different concepts. The results indicate that they are related to poor nutritional status or intake. Loneliness is often defined as a subjective measure of the feeling of being isolated, while social isolation is an objective measure of inadequate social connectedness [44]. On the other hand, social support refers to the provision of different types of assistance to others, which can alleviate social isolation and loneliness [45]. In two of the included studies, social support was measured using the Social Support Rating Scale [46], which contains the sub-domains of subjective support, support utilization, and objective support. In the study by Li et al. [30], an analysis of the sub-domains was conducted, and it was found that low subjective support and low support utilization were associated with low muscle mass, to which inadequate daily protein intake is often a contributor; while no such association was found with low objective support. This suggests that the subjective perception of being supported might be more important than the actual receipt of support.

Social frailty is an underexplored and emerging concept in the literature [39]. It was not included in the other review articles on social factors and nutritional status or dietary intake [9,10,11,12]. Frailty describes the vulnerability related to ageing. Some models stress the physical aspects of frailty, while others view frailty as a multidimensional concept, which can be divided into physical, psychological, and social aspects [39]. In a scoping review, social frailty was defined as the vulnerability of losing resources to fulfil one or more basic social needs across a lifespan [39]. The components of social frailty include social resources [39], social activities [39,47], social network [47], and general resources [39]. The sociological indicators of social frailty include living alone, social isolation, a low frequency of social activities, loneliness, a lack of social support, and a reduced social network. Eating alone, on the other hand, was shown to be a risk factor of social disengagement for Chinese older adults [36], and also for Japanese older adults [48]. The concept of social frailty fits with the theoretical framework proposed by Payette et al. [6], in which social frailty can affect the social aspects of the purchase, preparation, and consumption of food, which will affect the nutritional intake of the older population.

The results from the scoping review showed that other than physical and psychological factors, social factors including marital status, living and eating arrangement, social frailty, loneliness, social support, and social isolation may also play a significant role in contributing to malnutrition in community-dwelling older adults. Therefore, on top of conventional interventions that mainly address the physical and psychological aspects of nutrition in older adults, interventions that address modifiable social factors should be devised to ensure that a holistic approach is taken to effectively tackle malnutrition in older adults. Sahyoun et al. constructed a framework for designing a nutrition education intervention, and also suggested that consideration be given to modifying individual, social, and physical aspects of nutrition to promote changes in behaviour [49].

There are a few documented nutritional interventions that could also provide social support in modern Western countries. For example, congregate meal services in the United States provide community-dwelling older adults with healthy meals and social support [50]. Social bonds can also be facilitated by meal delivery services between delivery staff and recipients as the delivery staff hands the meals to the service users [51]. However, further investigations will be needed to determine if these interventions can be effective for the Chinese older population due to the collectivistic culture, where social support from people they identify with (in-group) is preferred over others (out-group) [52]. Therefore, interventions delivered by peers may allow development of longer-term friendship and provision of effective social support for community-dwelling Chinese older adults [45,53,54].

### Strengths and Limitations

This scoping review was the first to explore social factors of nutritional intake or status in Chinese older adults, and to include articles written in both English and Chinese. The implications of the review might be transferrable to other cultures that also value commensality during meals. However, this review has some limitations. Only a limited number of studies were included; hence, there were only one or two studies on most social factors. In some studies, the relationship between social factors and dietary intake/nutritional status was not investigated as the aim or the primary outcome. The factors might not have been adequately explored. All reviewed studies were cross-sectional or cohort studies, hence the causal relationship between social factors and dietary intake/nutritional status could not be established.

## 5. Conclusions

This scoping review showed that the relationship between social factors and dietary intake or nutritional status in community-dwelling Chinese older adults has not been extensively studied. There was no documented social intervention for improving dietary intake or nutritional status. Nonetheless, the social factors of being single, eating alone, being lonely, low social support, social isolation, and being socially frail could be associated with poorer dietary intake as measured by quantity or quality of dietary intake, or nutritional status as measured by anthropometry, biomarker, and tools for nutritional risks. Among these factors, having company during mealtimes, loneliness, and social frailty are modifiable. For future research designs, the use of qualitative studies can add to the richness of the data. Multifactorial interventions such as peer support programmes targeting loneliness, different aspects of social frailty, and company during mealtimes can also be implemented alongside conventional interventions to address malnutrition in community-dwelling Chinese older adults holistically.

## Figures and Tables

**Table 1:** Search terms for database searching.

Population			Social Factors	Dietary Intake/Nutritional Status
Community-dwelling Independent living Free-living	Chinese	Aged Old* Elder* Senior* Geriatric*	Social factors Social interaction Social isolation Social support Home environment Loneliness Alone Friends Companion* Commensal*	Nutritional status Malnutrition Undernutrition Nutrition* Diet* Eating Body weight Body Mass Index Body mass BMI Food Meals

Note: Search terms within the same column were combined with ‘OR’ and search terms among different columns were combined with ‘AND’.

**Table 2:** Characteristics of the included studies.

Reference	Origin	Aims	Study Design	Population	Measures of Dietary Intake/Nutritional Status	Measures of Social Factors	Key Findings Related to Nutritional Status and Social Factors
Bian et al. (2023) [27]	China	To explore the correlation between social support, sarcopenia, and cognitive impairment in Chinese older adults	Cross-sectional	720 Chinese community-dwelling older adults aged ≥65 years	Sarcopenia	Social Support Rate Scale	There was a negative association between older adults with higher levels of social support and sarcopenia.
Cui et al. (2024) [28]	China	To develop a nomogram for predicting the risk of AA among older people	Cross-sectional	2144 community-dwelling older adults aged ≥60 years in Jinan city	Simplified Nutritional Appetite Questionnaire	UCLA Loneliness Scale, living arrangement	Lonely older adults were more likely to have AA than non-lonely older adults (OR = 1.930). Older adults living alone were more likely to have AA than those who were not living alone (OR = 1.649).
Han et al. (2009) [29]	China	To examine the levels of nutritional and functional status; to identify the relationships between nutrition, demographic characteristics, and functional status; and to explore the predictors contributing to poor nutrition for older adults	Cross-sectional	162 community-dwelling older adults aged ≥65 years in Wuhan	MNA	Marital status, living arrangement	Widowed participants were more likely to suffer from poor nutrition. Living arrangement was not associated with nutritional status.
Huang et al. (2017) [38]	Taiwan	To evaluate whether daily frequency of eating with others is associated with all-cause mortality	Cohort study	1984 free-living Taiwanese older men and women aged ≥65	BMI, FFQ, DDS (calculated from a 24-h recall)	Eating arrangement	Female participants who did not eat with others had the lowest BMI. Male participants who ate only once daily with others had the lowest DDS. Female participants who did not eat with others had the lowest DDS. Male participants who did not eat with others ate the most meat and the least vegetables. Female participants who did not eat with others ate the least meat, least seafood, fewest eggs, and least vegetables.
Li et al. (2024) [30]	China	To investigate longitudinal associations between overall social support and its sub domains with the risk of sarcopenia	Cohort study	1905 community-dwelling adults aged ≥50 years in west China	Sarcopenia	Social Support Rating Scale	Participants with higher scores for overall social support, subjective support, and support utilization at baseline were less likely to develop sarcopenia during the 5-year follow up (adjusted OR = 0.87, 0.88, and 0.87, respectively).
Pek et al. (2020) [36]	Singapore	To understand the impact of social frailty on pertinent outcomes	Cross-sectional	229 community-dwelling adults aged ≥50 years (mean age 67.22; 92.6% Chinese)	MNA, serum albumin level	Social frailty	Socially frail participants had the lowest MNA scores. Socially pre-frail participants had the lowest serum albumin level. Socially frail participants were more likely to have poor nutritional status (adjusted OR = 8.35).
Song et al. (2020) [31]	China	To investigate the prevalence of multidimensional frailty, and to explore the relationship of general and abdominal obesity to multidimensional frailty in community-dwelling older people with hypertension	Cross-sectional	995 community-dwelling Chinese older people with hypertension aged ≥65 years	Consumption of breakfast, BMI, WC	Social frailty	Participants who did not have breakfast scored higher in social frailty. Overweight or obese participants scored higher in social frailty. Centrally obese participants scored higher in social frailty.
Tang et al. (2023) [32]	China	To evaluate the risk of malnutrition among the community-dwelling elderly and explore its influencing factors	Cross-sectional	950 seniors aged ≥65 years in Shanghai	Risk Assessment of Malnutrition in the Elderly	Marital status, living arrangement	Unmarried seniors were more likely to have malnutrition (OR = 1.755). Seniors living alone were more likely to have malnutrition (OR = 2.28).
Wang et al. (2013) [33]	China	To understand the physical and psychological health of older adults without social insurance, and to analyze their correlation and the factors affecting them	Cross-sectional	243 community-dwelling females aged ≥60 and over without social insurance in Beijing	BMI, WC	UCLA Loneliness Scale	Higher BMI was associated with lower loneliness (no participants were underweight).
Wang et al. (2023) [34]	China	To investigate the relationship between social isolation, depression, nutritional status, and quality of life among community-dwelling older adults during COVID-19	Cross-sectional	406 community-dwelling older adults aged ≥ in Shanghai	Risk Assessment of Malnutrition in the Elderly	Lubben Social Network Scale	Social isolation was weakly associated with malnutrition risk (r = 0.14).
Wei et al. (2018) [35]	China	To provide a nationally representative estimate of the prevalence of malnutrition in elderly Chinese adults and to determine predictors of malnutrition in this population	Cross-sectional	6394 community-dwelling Chinese adults aged ≥ 0 years	ESPEN definition of malnutrition (BMI, weight loss)	Marital status	Married participants were less likely to be malnourished (OR = 0.54).
Yap et al. (2007) [37]	Singapore	To describe the responses to the DETERMINE checklist and the nutritional risk level of community-dwelling older Chinese in Singapore	Cross-sectional	2605 community-dwelling older Chinese aged ≥55 years (mean age 66.0)	DETERMINE (nutritional risk)	Marital status, living arrangement	Single/divorced/widowed participants were more likely to be at a higher nutritional risk (adjusted OR = 1.46). Participants who lived alone were more likely to be at a higher nutritional risk (adjusted OR = 2.05).

Note: AA: anorexia of ageing; BMI: body mass index; DDS: Diet Diversity Score; DETERMINE: Disease, Eating poorly, Tooth loss/mouth pain, Economic hardship, Reduced social contact, Multiple medicines, Involuntary weight loss/gain, Needs assistance in self-care, Elder years above age 80; ESPEN: European Society of Parenteral and Enteral Nutrition and Metabolism; FFQ: Food Frequency Questionnaire; MNA: Mini Nutritional Assessment; OR: odds ratio; WC: waist circumference.

**Table 3:** Assessing risk of bias using the JBI critical appraisal checklist for analytical cross-sectional studies.

Study	Risk of Bias Domains *								% Yes
	1	2	3	4	5	6	7	8	
Bian 2023 [27]	✓	✓	✓	✓	✓	✓	✓	✓	100
Cui 2024 [28]	✓	✓	✓	✓	✓	✓	✓	✓	100
Han 2009 [29]	✓	✓	✓	✓	✓	✓	✓	✓	100
Pek 2020 [36]	✓	✓	?	✓	✓	✓	✓	✓	87.5
Song 2020 [31]	✓	✓	✓	✓	✓	✓	✓	✓	100
Tang 2023 [32]	✓	✓	✓	✓	✓	✓	✓	✓	100
Wang 2013 [33]	?	✓	✓	✓	✓	✓	✓	✓	87.5
Wang 2023 [34]	✓	✓	✓	✓	✓	✓	✓	✓	100
Wei 2018 [35]	✓	✓	✓	✓	✓	✓	✓	✓	100
Yap 2007 [37]	✓	✓	✓	✓	✓	✓	✓	✓	100

* Domains: 1—clearly defined inclusion criteria; 2—detailed description of study subjects and setting; 3—valid and reliable measurement of exposure; 4—objective and standardized measurement of condition; 5—identification of confounding factors; 6—control of confounding factors; 7—valid and reliable measurement of outcome; 8—use of appropriate statistical analysis; ✓—yes; ?—unclear.

**Table 4:** Assessing risk of bias using the JBI critical appraisal checklist for cohort studies.

Study	Risk of Bias Domains *											% Yes
	1	2	3	4	5	6	7	8	9	10	11	
Huang 2017 [38]	✓	✓	✓	✓	✓	✓	✓	✓	✓	N/A	✓	100
Li 2024 [30]	✓	✓	✓	✓	✓	✓	✓	✓	✓	✓	✓	100

* Domains: 1—groups recruited from same population; 2—exposure measured similarly between groups; 3—valid and reliable measurement of exposure; 4—identification of confounding factors; 5—control of confounding factors; 6—subjects free of outcome at baseline; 7—valid and reliable measurement of outcome; 8—reported and sufficient follow-up time for outcomes to occur; 9—complete follow-up or described reasons to loss to follow-up; 10—use of strategies to address incomplete follow-up; 11—use of appropriate statistical analysis; ✓—yes; N/A—not applicable.

**Table 5:** Summary of included studies showing relationships between social factors and dietary intake/nutritional status.

	No Significant Associations	Associated with Poor Dietary Intake/Nutritional Status
Single *		[29,35,37]
Living alone	[29]	[28,32,37]
Eating alone		[38]
Loneliness/low social support/social isolation		[27,28,30,33,34]
Social frailty		[31,36]

* Includes those who were unmarried, divorced, or widowed.

## Data Availability

All data related to this research are available within the manuscript.

## Supplementary Materials

The following supporting information can be downloaded at https://www.mdpi.com/article/10.3390/nu17122019/s1, Table S1. Syntax of PubMed search strategy.

## Abbreviations

The following abbreviations are used in this manuscript:

MNA	Mini Nutritional Assessment
AA	Anorexia of ageing
BMI	Body mass index
DDS	Diet Diversity Score
DETERMINE	Disease, Eating poorly, Tooth loss/mouth pain, Economic hardship, Reduced social contact, Multiple medicines, Involuntary weight loss/gain, Needs assistance in self-care, Elder years above age 80
ESPEN	European Society of Parenteral and Enteral Nutrition and Metabolism
FFQ	Food Frequency Questionnaire
OR	Odds ratio
WC	Waist circumference
