# Supplementary material for: Social Factors, Dietary Intake and the Nutritional Status of Community-Dwelling Chinese Older Adults: A Scoping Review
Source: Nutrients. 2025 Jun 17;17(12):2019. doi: 10.3390/nu17122019 (PMC12195862; doi:10.3390/nu17122019)
Supplement: Supplementary file 1 [file nutrients-17-02019-s001.zip › nutrients-3670449-supplementary.pdf]

Table S1. Syntax of PubMed search strategy

1	"independent living"[MeSH Terms] OR ("independent"[All Fields] AND "living"[All Fields]) OR "independent living"[All Fields] OR ("independent living"[MeSH Terms] OR ("independent"[All Fields] AND "living"[All Fields]) OR "independent living"[All Fields] OR ("community"[All Fields] AND "dwelling"[All Fields]) OR "community dwelling"[All Fields]) OR ("free"[All Fields] AND ("lived"[All Fields] OR "lives"[All Fields] OR "living"[All Fields] OR "livings"[All Fields]))
2	"chineses"[All Fields] OR "east asian people"[MeSH Terms] OR ("east"[All Fields] AND "asian"[All Fields] AND "people"[All Fields]) OR "east asian people"[All Fields] OR "chinese"[All Fields]
3	"old"[All Fields] OR ("older"[All Fields] OR "olders"[All Fields]) OR "elder*"[All Fields] OR "senior*"[All Fields] OR "geriatric*"[All Fields]
4	"social factors"[MeSH Terms] OR ("social"[All Fields] AND "factors"[All Fields]) OR "social factors"[All Fields]) OR ("social interaction"[MeSH Terms] OR ("social"[All Fields] AND "interaction"[All Fields]) OR "social interaction"[All Fields]) OR ("social isolation"[MeSH Terms] OR ("social"[All Fields] AND "isolation"[All Fields]) OR "social isolation"[All Fields]) OR ("social support"[MeSH Terms] OR ("social"[All Fields] AND "support"[All Fields]) OR "social support"[All Fields]) OR ("home environment"[MeSH Terms] OR ("home"[All Fields] AND "environment"[All Fields]) OR "home environment"[All Fields]) OR ("lonelier"[All Fields] OR "loneliness"[MeSH Terms] OR "loneliness"[All Fields]) OR ("home environment"[MeSH Terms] OR ("home"[All Fields] AND "environment"[All Fields]) OR "home environment"[All Fields] OR "alone"[All Fields]) OR ("friend s"[All Fields] OR "friending"[All Fields] OR "friends"[MeSH Terms] OR "friends"[All Fields] OR "friend"[All Fields]) OR "companion*"[All Fields] OR "commensal*"[All Fields]
5	"nutritional status"[MeSH Terms] OR ("nutritional"[All Fields] AND "status"[All Fields]) OR "nutritional status"[All Fields] OR ("malnutrition"[MeSH Terms] OR "malnutrition"[All Fields] OR "malnutrition s"[All Fields] OR "malnutritional"[All Fields] OR "malnutritions"[All Fields]) OR ("malnutrition"[MeSH Terms] OR "malnutrition"[All Fields] OR "undernutrition"[All Fields] OR "undernutritional"[All Fields]) OR "nutrition*"[All Fields] OR "diet*"[All Fields] OR ("eating"[MeSH Terms] OR "eating"[All Fields]) OR ("body weight"[MeSH Terms] OR ("body"[All Fields] AND "weight"[All Fields]) OR "body weight"[All Fields]) OR ("body mass index"[MeSH Terms] OR ("body"[All Fields] AND "mass"[All Fields] AND "index"[All Fields]) OR "body mass index"[All Fields]) OR (("human body"[MeSH Terms] OR ("human"[All Fields] AND "body"[All Fields]) OR "human body"[All Fields] OR "body"[All Fields]) AND ("molecular weight"[MeSH Terms] OR ("molecular"[All Fields] AND "weight"[All Fields]) OR "molecular weight"[All Fields] OR "mass"[All Fields])) OR "BMI"[All Fields] OR ("food"[MeSH Terms] OR "food"[All Fields]) OR ("meal s"[All Fields] OR "meals"[MeSH Terms] OR "meals"[All Fields])
6	1 AND 2 AND 3 AND 4 AND 5
